# A novel scale to predict acute anterior circulation large vessel occlusion stroke for community hospitals: result from the STRESS registry

**DOI:** 10.3389/fneur.2026.1776311

**Published:** 2026-03-27

**Authors:** Weiwen Yi, Anhua Li, Yuemei He, Wei Lv, Xinghang Lan, Yan Liu, Geng Liao

**Affiliations:** 1 Department of Neurology, Maoming School of Clinical Medicine, Guangdong Medical University (Maoming People’s Hospital), Maoming, China; 2 Department of Disease Prevention, Maoming School of Clinical Medicine, Guangdong Medical University (Maoming People’s Hospital), Maoming, China; 3 Department of Health Care, The Second Medical Center, Chinese PLA General Hospital, Beijing, China

**Keywords:** anterior circulation, etiology, large vessel occlusion, scale, stroke

## Abstract

**Objective:**

To develop a simple and novel scale to predict acute anterior circulation large vessel occlusion (LVO) strokes for community hospitals in China.

**Methods:**

We conducted a retrospective analysis of a prospectively collected acute ischemic stroke patient database (STRESS registry). The patients were divided into the derivation and validation cohorts. We derived a scale in the derivation cohort and assessed the scale in the validation cohort.

**Results:**

A total of 1,196 patients were screened for acute ischemic stroke, 722 patients were included to the study, 406 and 316 in the derivation and validation cohorts, respectively. Multivariable logistic regression analysis identified gaze deviation [odds ratio (OR): 5.66, 95% confidence interval (CI): 2.79–11.50], motor arm weakness (OR: 4.17, 95% CI: 1.17–14.87), atrial fibrillation (OR: 4.10, 95% CI: 2.12–7.90), and level of consciousness (OR: 3.76, 95% CI: 1.56–9.04) were significantly associated with acute anterior circulation large vessel occlusion stroke. Therefore, a four-item community hospitals stroke scale was developed, including consciousness, eye gaze, arm weakness, and atrial fibrillation (CEA^2^). In the validation cohort, the Youden index, sensitivity, specificity, positive predictive value, negative predictive value, and accuracy of CEA^2^ ≥ 2 in predicting anterior circulation LVO strokes were 0.722, 0.878 (95% CI 0.818–0.921), 0.844 (95% CI 0.745–0.865), 0.846 (95% CI 0.755–0.871), 0.876 (95% CI 0.809–0.917), and 0.861 (95% CI 0.801–0.881), respectively. Further analysis revealed that when the CEA^2^ score, including atrial fibrillation, was ≥ 2, the sensitivity, specificity, and accuracy for predicting cardiogenic embolism etiology were 0.792(95% CI 0.722–0.850), 0.849 (95% CI 0.785–0.899), and 0.821 (95% CI 0.773–0.862), respectively.

**Conclusion:**

The CEA^2^ scale may be a simple and effective tool that identifies anterior circulation LVO strokes and provides relevant etiology information for community hospitals in China.

## Introduction

Large vessel occlusion (LVO) stroke is often associated with significant disability and high mortality rates. The current guidelines for the early management of acute ischemic stroke (AIS) recommend endovascular treatment as favorable for selected acute anterior circulation large vessel occlusion stroke patients. However, the effectiveness of endovascular procedures is critically dependent on timely intervention (1–3). In cases of large vessel occlusions, approximately 70%–80% of lesions primarily occur in the anterior circulation, predominantly distributed in the middle cerebral artery and internal carotid artery (4). Research has indicated that LVO may be present when the National Institutes of Health Stroke Scale (NIHSS) score exceeds 9–14 points (5–7). However, despite its strong discriminatory power, the NIHSS is complex and time-consuming for emergency medical services (EMS) to administer. To address this, several simplified scales have been developed (8–12). These scales were designed using various methods, including expert-based screening, statistical approaches, or a combination of both. Notably, some studies validated RACE (Rapid arterial occlusion evalutation scale) may be a better performing stroke scales than the others (13). However, the RACE remains a complex scale for community physicians,especially in China. Therefore, there is an urgent need for a simple, user-oriented scale that can detect cortical or hemispheric functional deficits to improve the prediction of acute anterior circulation LVO strokes for community hospitals in China.

## Methods

### Patient

We did a retrospective analysis from the pool of STRESS study (STroke REgiStry program in Yuexi District of China, Chinese Clinical Trial Registry, http://www.chictr.org.cn; ChiCTR2100052952) between June 2022 and June 2024. STRESS study is an ongoing, prospective, multicenter, real-world stroke registry clinical study conducted in 15 comprehensive stroke centers in China, and launched in January 2022. The study protocol are listed in the Supplementary Material S1.

Our study has been approved by the Ethics Review Committee of these hospitals and adheres to the principles of the Helsinki Declaration. We included patients who met the following criteria: (1) diagnosis of acute ischemic stroke confirmed by computed tomography or magnetic resonance imaging within 24 h of onset; (2) age ≥ 18 years; (3) occluded vessel was a responsible vessel in the anterior circulation; and (4) had a detailed NIHSS score and medical history at admission. The exclusion criteria included the following: (1) time from onset to treatment > 24 h; (2) history of stroke with residual neurological deficits, defined as a modified Rankin Scale score greater than 2 prior to the stroke; (3) presence of other conditions leading to motor or sensory impairment; (4) severe heart, liver, or kidney dysfunction; (5) allergy to iodinated contrast agents or other reasons preventing completion of neck and head computed tomography angiography (CTA), magnetic resonance angiography (MRA), or digital subtraction angiography (DSA) examinations; (6) with infarction in the posterior circulation; and (7) incomplete NIHSS score or other key records. The definition of acute anterior circulation LVO strokes is based on unilateral occlusion of the intracranial segment of the internal carotid artery or the M1/M2 segments of the middle cerebral artery as detected by CTA, MRA, or DSA. According to the inclusion and exclusion criteria, patients were consecutively enrolled and divided into derivation and validation cohorts based on chronological order of admission. Two experienced neurologists assessed the occlusion status while maintaining patient confidentiality, and any discrepancies in their evaluations were resolved through consensus.

### Data collection and definition

Patient demographic characteristics, comorbidities, premorbid functional status, conventional vascular risk factors, NIHSS score at presentation, arterial occlusion site, etiology, and results of brain imaging and laboratory assessments were collected. Current smokers were those who smoked cigarettes or other tobacco products or quit in the last year (14). Alcohol consumers were defined as those consuming an average of > 7 standard drinks weekly (15). The presence of atrial fibrillation (AF) included a history of paroxysmal, persistent, and permanent AF, as well as newly diagnosed AF detected on an electrocardiogram obtained in the ambulance or upon admission. The location of the initial occlusion site was determined at baseline using CTA or MRA and DSA. NIHSS scores were recorded by neurologists with more than 5 years of clinical experience in the STRESS study.

### Derivation cohort

Patients with anterior circulation ischemic stroke were divided into the LVO and non-LVO stroke cohorts based on the presence or absence of anterior LVO strokes in the derivation cohort. Data were collected, including patient demographic characteristics, comorbidities, premorbid functional status, conventional vascular risk factors, NIHSS scores at presentation, arterial occlusion site, etiology, and results of brain imaging and laboratory assessments. Differences in these indicators between the LVO and non-LVO stroke cohorts were compared, and binary logistic regression analysis was performed to identify independent predictors of LVO strokes. According to clinical practice and expert consensus, the screening tool needs to be simple and user-oriented; therefore, the maximum number of items in the new scale was set to 4. Subsequently, receiver operating characteristic (ROC) curve analysis was conducted to determine the predictive values of different combinations of these items that are highly correlated with anterior circulation LVO strokes. Based on the analysis results, a new stroke scale was developed.

### Validation cohort

The validation cohort to retrospectively validate the scale. The sensitivity, specificity, positive predictive value, negative predictive value, and accuracy for predicting anterior circulation LVO strokes were calculated. The primary objective of this study was to assess the accuracy of the new stroke scale in detecting true candidates for LVO strokes in the validation cohort.

### Statistical analysis

Patients were divided into the derivation and validation cohorts, with each cohort containing the LVO and non-LVO stroke groups. Statistical analysis of the research data was performed using SPSS 26.0. Non-normally distributed continuous variables were expressed as median (Q1, Q3) and compared using the Wilcoxon rank-sum test. Categorical data were presented as counts and percentages, with comparisons made using the chi-square test. Normally distributed measurement data were expressed as mean ± standard deviation, and intercohort comparisons were conducted using t tests. Based on the factors associated with LVO strokes identified in univariate analysis, binary logistic regression was used to screen for independent risk factors for LVO strokes. ROC curves were plotted. The area under the curve (AUC) and Youden index were calculated to determine the optimal diagnostic cutoff of the new community hospitals stroke scale for LVO strokes. To assess potential overfitting and optimism in model performance, we performed bootstrap internal validation with 200 resamples in the derivation cohort. To evaluate potential multicollinearity among the candidate predictors, we calculated Pearson correlation coefficients and variance inflation factors (VIF). A correlation coefficient >0.8 or VIF > 10 was considered indicative of problematic collinearity. To further validate the robustness of predictor selection, we performed least absolute shrinkage and selection operator (LASSO) logistic regression with 10-fold cross-validation. The optimal regularization parameter *λ* was selected using the minimum deviance criterion (λ.min) and the one standard error rule (λ.1se). Variables with non-zero coefficients at λ.1se were considered as final selected predictors. Calibration of a model was assessed using calibration plots with 200 bootstrap resamples for optimism correction, as implemented in the “rms” package. The calibration intercept (calibration-in-the-large) and calibration slope were computed using the “val.prob” function. The Brier score was calculated as the mean squared difference between predicted probabilities and observed outcomes. Decision curve analysis (DCA) was performed using the rmda package in R. Net benefit was calculated across threshold probabilities from 0 to 1 (increments of 0.01) for the full logistic model.

## Results

### Baseline characteristics

**Figure 1:**
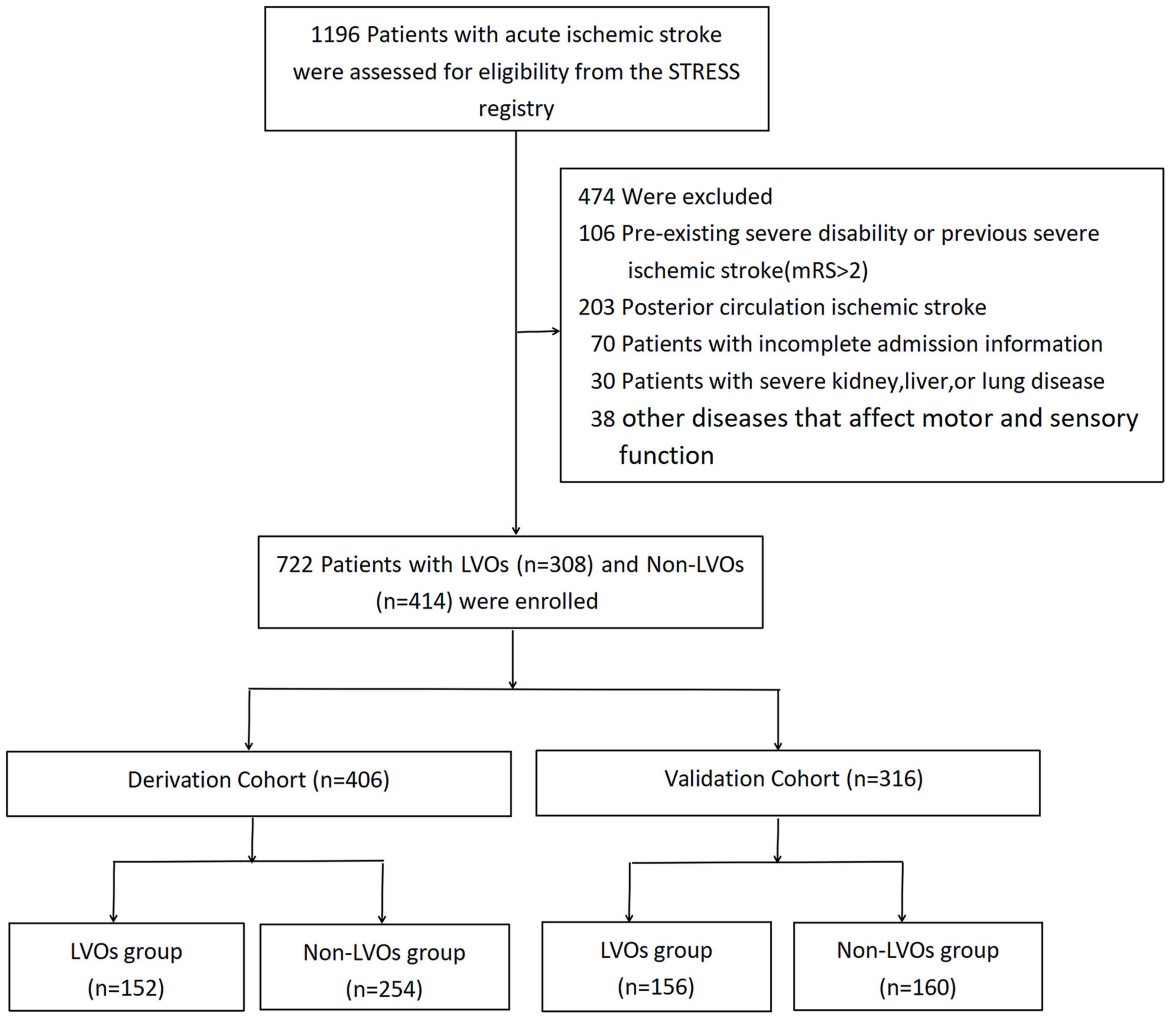
Flow chart. NIHSS, National Institutes of Health Stroke Scale; LVOs, large vessel occlusive stroke.

A total of 1,196 patients were screened for acute ischemic stroke, 722 patients were included to the study. Consecutive patients enrolled between June 2022 and March 2023 constituted the derivation cohort (*n* = 406), and those enrolled between April 2023 and June 2024 formed the validation cohort (*n* = 316). This study flow chart was shown in Figure 1. In the derivation cohort, the baseline demographic characteristics between LVO and non-LVO stroke patients exhibited no significant differences in age or gender the mean age was similar between non-LVO (69.51 ± 13.22 years) and LVO stroke patients (69.09 ± 12.22 years, *p* = 0.750). Likewise, the percentage of male patients was comparable (60.24 vs. 61.18%, *p* = 0.850) (Supplementary Table S1). These trends were consistent in the validation cohort, where neither age nor gender exhibited statistical significance between cohorts (*p* = 0.563 and *p* = 0.201, respectively). However, a marked difference was observed in the prevalence of AF, which was significantly higher in LVO stroke patients in both cohorts. In the derivation cohort(Supplementary data), AF was present in 48.03% of LVO stroke patients compared with only 9.84% in non-LVO stroke patients (*p* < 0.001), and this pattern held in the validation cohort (46.79 vs. 8.12%, *p* < 0.001) (Supplementary Table S2). This difference underscores AF as a critical factor associated with LVO strokes. The baseline demographic and clinical characteristics of the two cohorts were shown in Supplementary Table S3.

The stroke severity measured using the NIHSS revealed substantial differences between the two cohorts. In the derivation cohort, LVO stroke patients had a significantly higher median NIHSS score (median 12.00, IQR 9.00–16.00) than non-LVO stroke patients (median 4.00, IQR 2.00–6.00, *p* < 0.001), a trend that was also evident in the validation cohort (13.00 vs. 4.00, *p* < 0.001). In the derivation and validation cohorts, univariate analysis revealed significant differences between LVO and non-LVO stroke patients in the level of consciousness (LOC), LOC questions, LOC commands, gaze deviation, facial palsy, motor arm, motor leg, and aphasia (Supplementary Tables S1, S2).

### CEA^2^ scale

**Figure 2:**
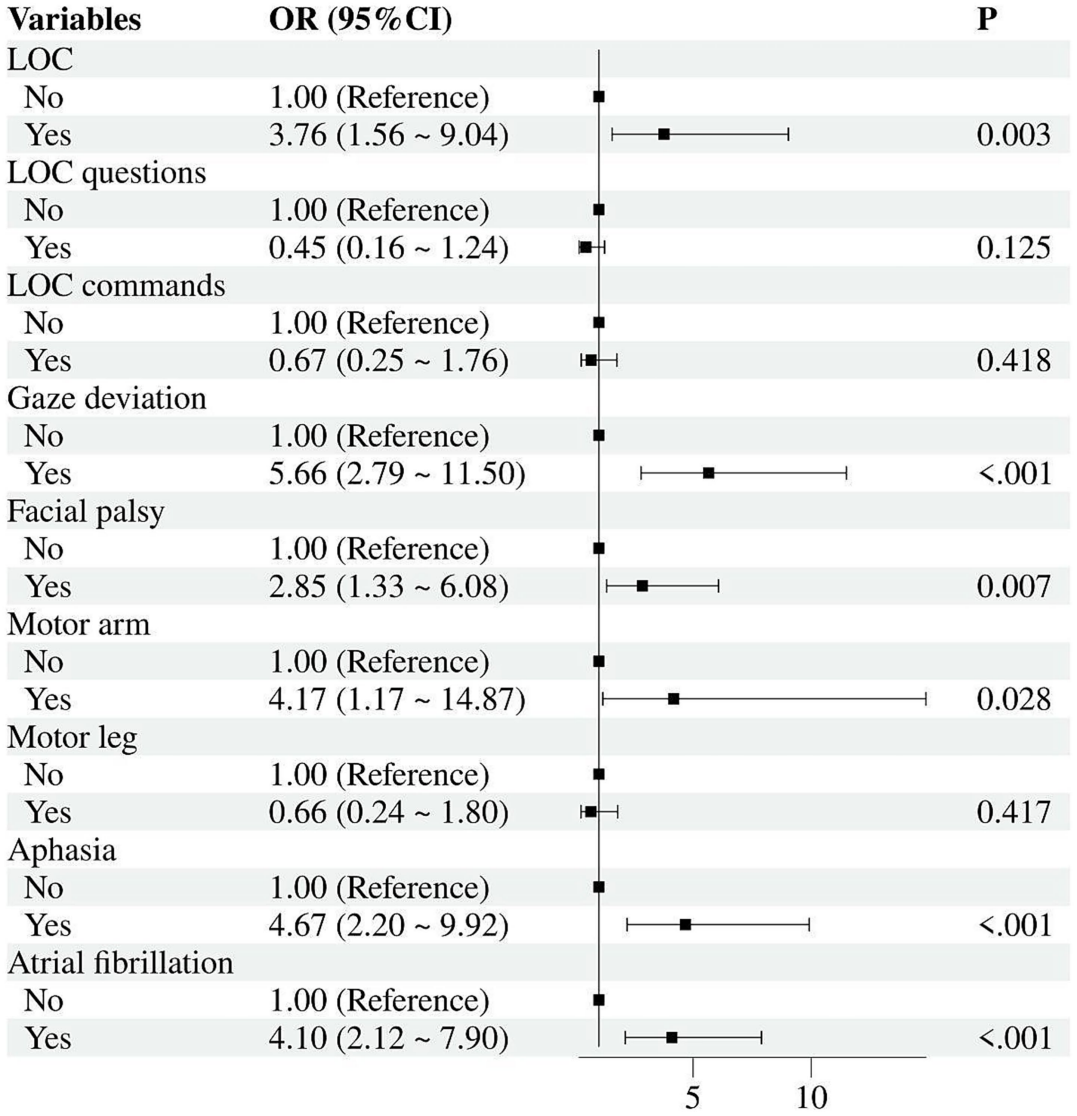
Multivariable logistic regression models of the clinical factors associated with LVOs derivation cohort. LOC, Level of Consciousness; OR, Odds Ratio; CI, Confidence Interval.

Several clinical factors were significantly associated with LVO strokes based on multivariable logistic regression analysis (Figure 2). Gaze deviation was identified as the strongest predictor, with an odds ratio (OR) of 5.66 (95% confidence interval [CI]: 2.79–11.50, *p* < 0.001). Other notable factors included aphasia (OR: 4.67, 95% CI: 2.20–9.92, *p* < 0.001), motor arm (OR: 4.17, 95% CI: 1.17–14.87, *p* = 0.028), AF (OR: 4.10, 95% CI: 2.12–7.90, *p* < 0.001), LOC (OR: 3.76, 95% CI: 1.56–9.04, *p* = 0.003), and facial palsy (OR: 2.85, 95% CI: 1.33–6.08, *p* = 0.007). These findings indicate that gaze deviation, aphasia, motor arm, AF, LOC, and facial palsy are key predictors of LVO strokes. Although aphasia is closely related to LVO strokes, this item was excluded to avoid difficulties and inconsistencies for healthcare personnel during the assessment. Using the multiple logistic regression models, the consciousness –eye gaze–arm weakness–atrial fibrillation (CEA^2^) scale was derived to facilitate the prediction of LVO strokes. The integer point score for each predictor was derived by dividing its regression coefficient (*β*) by the smallest coefficient among the four variables (*β* = 1.22 for AF) and rounding to the nearest whole number (Supplementary Table S4). This yielded the following weights: level of consciousness = 1 (1.27/1.22 ≈ 1.04, assigned 1 point), gaze deviation = 2 (1.84/1.22 ≈ 1.51,assigned 2 points), arm weakness = 1 (1.48/1.22 ≈ 1.21, assigned 1 point), and atrial fibrillation = 1 (1.22/1.22 = 1.00,assigned 1 point). The total CEA^2^ score was calculated as the sum of these points (range 0–5). The components of the scale are described in Table 1. DCA comparing the full logistic model and the simplified CEA^2^ score indicated equivalent clinical utility in Supplementary Figure S1.

**Table 1:** The CEA^2^ scale and its correspondence to the NIHSS.

Item	CEA^2^	NIHSS
Consciousness		
Normal	0	0
Mild or severe disturbance	1	1–3
Eye		
Normal	0	0
Gaze preference, forced gaze deviation, or abnormal horizontal eye movements	2	1–2
Arm		
Normal or drift but does not hit bed	0	0–1
Some resistance to gravity, no resistance to gravity, or absence of movement	1	2–4
Atrial fibrillation		
No	0	
Yes	1	

CEA^2^, Consciousness-Eye-Arm-Atrial Fibrillation; AF, atrial fibrillation; NIHSS, National institutes of health stroke scale.

### ROC curve of the derivation and validation cohorts

**Figure 3:**
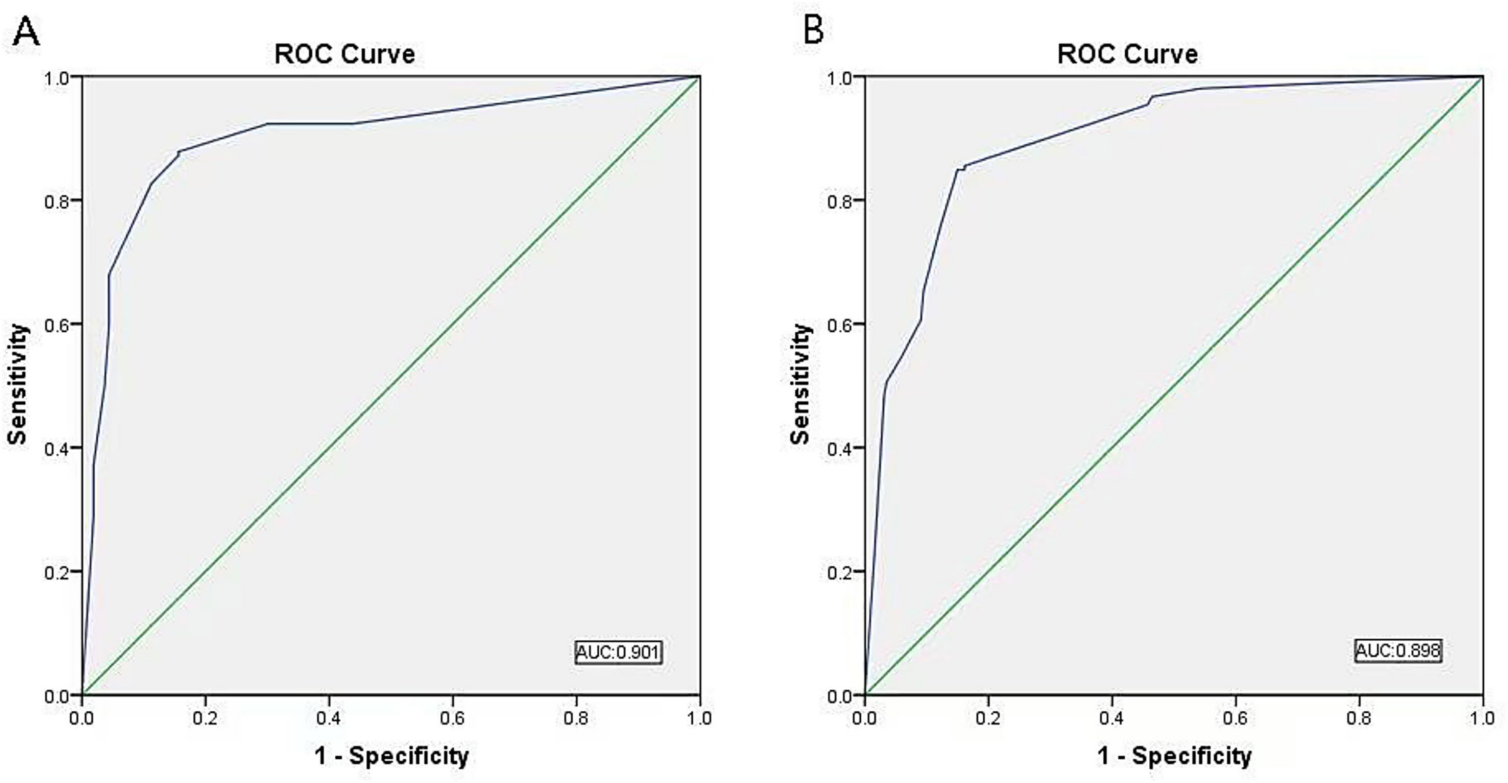
ROC of the CEA^2^ scale. **(A)** The validation cohort. **(B)** The derivation cohort. ROC, Receiver Operating Characteristic curve; CEA^2^, Consciousness-Eye-Arm-Atrial Fibrillation scale.

The ROC curves in Figure 3 illustrate the diagnostic performance of the validation and derivation cohorts in predicting large vessel occlusion in the anterior circulation. The validation cohort had an AUC of 0.901 (95% CI 0.835–0.906), whereas the derivation cohort had an AUC of 0.898 (95% CI 0.856–0.927). These AUC values shown in Figure 3.

### Diagnostic performance of CEA^2^ cutoff values

In both the derivation and validation cohorts, the CEA^2^ scale demonstrated variations in sensitivity and specificity across different cutoff values. In the derivation cohort, when the cutoff value was ≥ 2, the CEA^2^ scale demonstrated a sensitivity of 0.836 (95% CI 0.771–0.886), specificity of 0.794 (95% CI 0.739–0.839), positive predictive value (PPV) of 0.723 (95% CI 0.654–0.782), negative predictive value (NPV) of 0.883 (95% CI 0.834—0.919), and accuracy of 0.810 (95% CI 0.769–0.846). The Youden index was 0.630. In the validation cohort, at the same cutoff of ≥ 2, the CEA^2^ scale achieved a sensitivity of 0.878 (95% CI 0.818–0.921), specificity of 0.812 (95% CI 0.745–0.865), PPV of 0.820 (95% CI 0.755–0.871), NPV of 0.872 (95% CI 0.809–0.917), and accuracy of 0.845 (95% CI 0.801–0.881). The Youden index was 0.691 the diagnostic performance at other cutoff values is detailed in Table 2.

**Table 2:** Different cutoff value of the CEA^2^ in detecting LVOs.

Cutoff value of CEA^2^ score	Youden index	Sensitivity	Specificity	PPV	NPV	Accuracy
Derivation cohort (*n* = 406) ≥1	0.437	0.980	0.457	0.519	0.975	0.653
≥2	0.694	0.855	0.839	0.760	0.906	0.845
≥3	0.557	0.651	0.906	0.805	0.813	0.810
≥4	0.472	0.507	0.965	0.895	0.766	0.793
≥5	0.214	0.230	0.984	0.897	0.681	0.702
Validation cohort (*n* = 316) ≥1	0.486	0.923	0.563	0.673	0.882	0.741
≥2	0.722	0.878	0.844	0.846	0.876	0.861
≥3	0.603	0.647	0.956	0.935	0.736	0.804
≥4	0.476	0.513	0.963	0.930	0.670	0.741
≥5	0.269	0.288	0.981	0.936	0.586	0.639

CEA^2^, Consciousness-Eye-Arm-Atrial Fibrillation; NPV, negative predictive value; PPV, positive predictive value; LVOs, large vessel occlusion stroke.

Further analysis revealed that when the CEA^2^ score, including AF, At the cutoff of ≥2, the sensitivity was 0.792 (95% CI 0.722–0.850), specificity 0.849 (95% CI 0.785–0.899), PPV 0.836 (95% CI 0.767–0.889), NPV 0.807 (95% CI 0.741–0.861), and accuracy 0.821 (95% CI 0.773–0.862) (shown in Table 3).

**Table 3:** Different cutoff values of the CEA^2^ in detecting cardiogenic embolism the validation group.

Cutoff value of CEA^2^ score	Sensitivity	Specificity	PPV	NPV	Accuracy
**≥2**	**0.792**	**0.849**	**0.836**	**0.807**	**0.821**
≥3	0.740	0.873	0.851	0.775	0.808
≥4	0.662	0.899	0.864	0.731	0.782
≥5	0.519	0.937	0.889	0.667	0.731

CEA^2^, Consciousness-Eye-Arm-Atrial Fibrillation scale; NPV, negative predictive value; PPV, positive predictive value. Bold values indicate the diagnostic performance metrics at the corresponding cutoff.

The apparent AUC of the CEA^2^ model in the derivation cohort was 0.881 (95% CI: 0.856–0.906). After bootstrap internal validation, the average optimism was 0.0045, yielding an optimism-corrected AUC of 0.876 (Supplementary Table S5). The Pearson correlation coefficients indicating no substantial multicollinearity and LASSO logistic regression confirm that each variable contributes to the prediction of LVOs (Supplementary Table S6 and Supplementary Figure S2). Calibration and decision curve analysis shown the mean absolute error of the calibration curve was 0.027,suggesting good overall model fit, and DCA demonstrated that the CEA^2^ model provided a positive net benefit across a wide range of threshold probabilities (approximately 0.1 to 0.9), outperforming both the “treat all” and “treat none” strategies. These findings support the clinical utility of the CEA^2^ scale for triage decisions in community hospital settings (Supplementary Figures S3, S4).

### Comparison of diagnostic performance among four scales

In the total cohort of 722 patients with acute ischemic stroke, we compared the predictive performance of the CEA^2^ scale (cutoff ≥2), the NIHSS (cutoff ≥7), the SAVE (the speech arm vision eyes scale, cutoff ≥2), and the RACE (cutoff ≥5) for anterior circulation LVO stokes. The diagnostic performance metrics at the predefined cutoffs are summarized in Table 4. The NIHSS exhibited the highest sensitivity (89.2, 95% CI: 85.2–92.4%), negative predictive value (90.4, 95% CI: 86.9%–93.3%), accuracy (83.5, 95% CI: 80.6%–86.2%), and Youden index (68.3). The CEA^2^ scale achieved a sensitivity of 85.7% (95% CI: 81.4%–89.4%) and a specificity of 80.1% (95% CI: 75.9%–83.9%), resulting in an accuracy of 82.5% (95% CI: 79.6–85.2%) and a Youden index of 65.8. Compared with RACE and SAVE, CEA^2^ showed higher sensitivity (85.7% vs. 82.5%) and specificity (80.1% vs. 77.6%). The area under the receiver operating characteristic curve (AUC) was 0.881 (95% CI: 0.856–0.906) for CEA^2^, 0.917 (95% CI: 0.897–0.938) for NIHSS, 0.778 (95% CI: 0.748–0.809) for SAVE, and 0.844 (95% CI: 0.815–0.873) for RACE (Table 5). DeLong tests revealed that the AUC of CEA^2^ was higher than that of SAVE (*p* < 0.001) and RACE (*p* = 0.0009), lower than that of NIHSS (*p* < 0.001).

**Table 4:** Diagnostic performance of four scales for predicting large vessel occlusion.

Metric*	CEA^2^ (≥2)	NIHSS (≥7)	SAVE (≥2)	RACE (≥5)
Sensitivity (%)	85.7 (81.4–89.4)	89.2 (85.2–92.4)	84.1 (79.6–88.0)	82.5 (77.9–86.6)
Specificity (%)	80.1 (75.9–83.9)	79.1 (74.8–83.0)	62.9 (58.0–67.6)	77.6 (73.3–81.6)
PPV (%)	76.9 (72.2–81.2)	76.8 (72.1–81.0)	63.7 (58.9–68.3)	74.1 (69.2–78.6)
NPV (%)	87.9 (84.1–91.0)	90.4 (86.9–93.3)	83.7 (79.0–87.6)	85.2 (81.1–88.6)
Accuracy (%)	82.5 (79.6–85.2)	83.5 (80.6–86.2)	72.2 (68.7–75.4)	79.8 (76.7–82.7)
Youden index	65.8	68.3	47.0	60.2

^*^Data are presented as point estimate (95% confidence interval). CEA^2^, Consciousness-Eye-Arm-Atrial Fibrillation scale; NIHSS, National Institutes of Health Stroke Scale; SAVE, the speech arm vision eyes scale; RACE, Rapid arterial occlusion evaluation scale; PPV, positive predictive value; NPV, negative predictive value.

**Table 5:** AUC and pairwise comparisons of four scales.

Scale	AUC (95% CI)	Comparison with CEA^2^	*p* value*
CEA^2^	0.881 (0.856–0.906)	—	—
NIHSS	0.917 (0.897–0.938)	CEA^2^ vs. NIHSS	<0.001
SAVE	0.778 (0.748–0.809)	CEA^2^ vs. SAVE	<0.001
RACE	0.844 (0.815–0.873)	CEA^2^ vs. RACE	0.0009

*p values were calculated using the DeLong test. AUC, Area under the curve; CEA2, Consciousness-Eye-Arm-Atrial Fibrillation scale; NIHSS, National Institutes of Health Stroke Scale; SAVE, the speech arm vision eyes scale; RACE, Rapid arterial occlusion evaluation scale; PPV, positive predictive value; NPV, negative predictive value.

## Discussion

In this study, we developed the CEA^2^ community hospitals stroke scale. The scale comprises four items-LOC, Gaze, Arm drift and AF, which predicts acute anterior circulation LVO strokes with high sensitivity and specificity in a AIS hospital registry sample. Our findings demonstrate that a CEA^2^ score of ≥ 2 correlates strongly with the presence of anterior circulation LVO strokes, achieving sensitivity and accuracy of 87.8 and 86.1%, respectively.

We limited scoring to binary (absent or present) assessments to avoid the added time and complexity that accompany gradations of severity (16). Numerous studies have indicated that AF serves as an independent risk factor for LVO strokes and can be readily assessed prior to hospitalization for evaluating LVO strokes (17–19), and the inclusion of AF as a key indicator can provide etiological information through straightforward assessments. Furthermore, Literature has showed that gaze deviation offers a higher predictive value for LVO strokes than other neurological symptoms (20). These findings may account for the relatively strong predictive capability of the CEA^2^ scale.

Differences from other pre-hospital stroke scales, The CEA^2^ scale is a LVO stroke identification tool primarily developed for community hospitals, which ordinarily have basic examination equipment such as CT and electrocardiogram. We had compared the new scale with NIHSS, RACE and SAVE head-to-head, findings that while the CEA^2^ scale does not surpass the comprehensive NIHSS in overall discriminative ability, it outperforms the SAVE scale and is comparable to the widely used RACE scale, with the added advantage of including atrial fibrillation as a predictor. The high specificity and acceptable sensitivity of CEA^2^ support its potential utility as a rapid screening tool for LVO in community hospitals settings. Therefore, this scale is not suitable for pre-hospital LVO strokes identification. The early recognition of LVO strokes is crucial for favour outcomes, particularly as treatment options such as thrombectomy are time-sensitive. We expect to apply CEA^2^ scale, emergency physicians in community hospitals can better identify LVO strokes and transfer them to comprehensive hospitals quickly for further thrombectomy treatment. Traditional scoring systems such as NIHSS and RACE scale, require neurologists—these personnel lacking in community hospitals of China—to conduct accurate assessments, which limits their promotion and application. The simplicity of the CEA^2^—requiring only a binary assessment of four key items—ensures that even nonprofessional rescuers can efficiently determine the necessity for urgent care. Implementing simple and scientifically sound scoring criteria may also facilitate training and adherence among emergency responders, further increasing the likelihood of timely stroke interventions.

However, our study has several limitations. First, the retrospective design may lead to bias, as patient data were sourced from hospitals of China, limiting its generalizability to other populations and settings. Second, the scale was developed with comprehensive hospitals database, which probably induces an overestimation of the screening performance of the scale. Third, in spite of all data were collected by trained neurologists using standardized definitions, and any discrepancies in imaging interpretation were resolved by consensus, this study did not formally assess interrater reliability for the NIHSS.

## Conclusion

The 4-item CEA^2^ score, which consists of level of consciousness, eye gaze, arm weakness, and atrial fibrillation, may be used to identify anterior circulation LVO strokes for community hospitals. Further external validation is warranted.

## Data Availability

The original contributions presented in the study are included in the article/Supplementary material, further inquiries can be directed to the corresponding authors.
