# Supplementary figures and images for: A novel scale to predict acute anterior circulation large vessel occlusion stroke for community hospitals: result from the STRESS registry
Source: Front Neurol. 2026 Mar 27;17:1776311. doi: 10.3389/fneur.2026.1776311 (PMC13065725; doi:10.3389/fneur.2026.1776311)

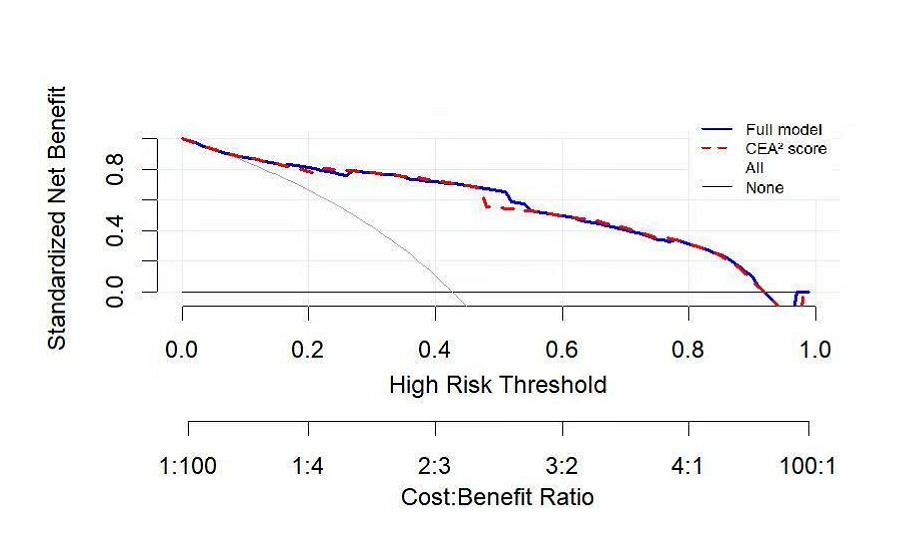

Supplement: Supplementary file 1 [file Image_1.tif]

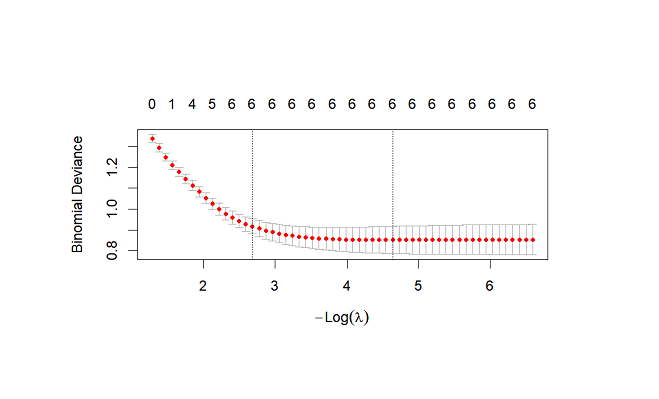

Supplement: Supplementary file 2 [file Image_2.tif]

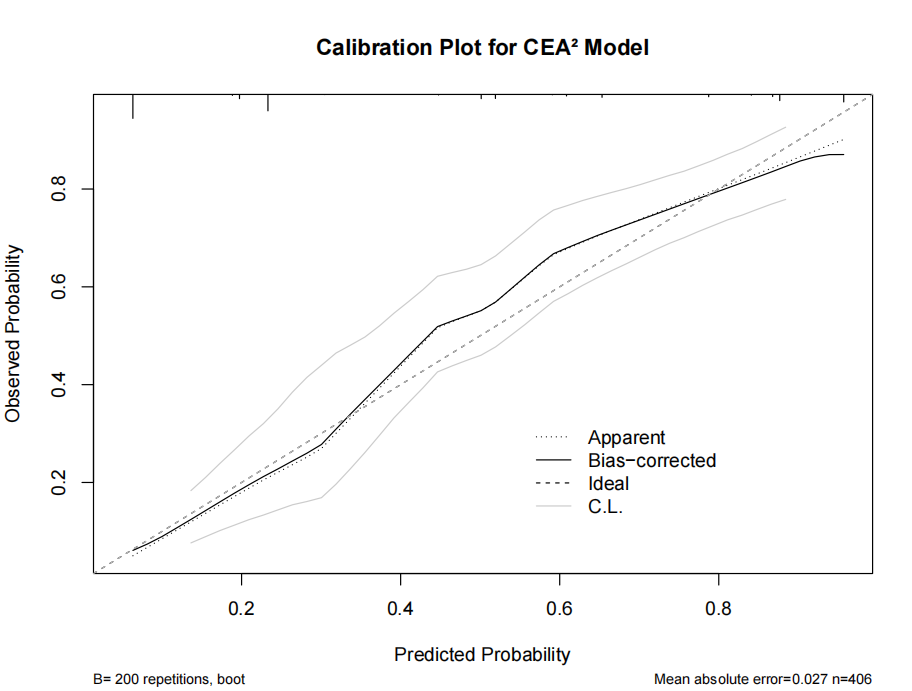

Supplement: Supplementary file 3 [file Image_3.tif]

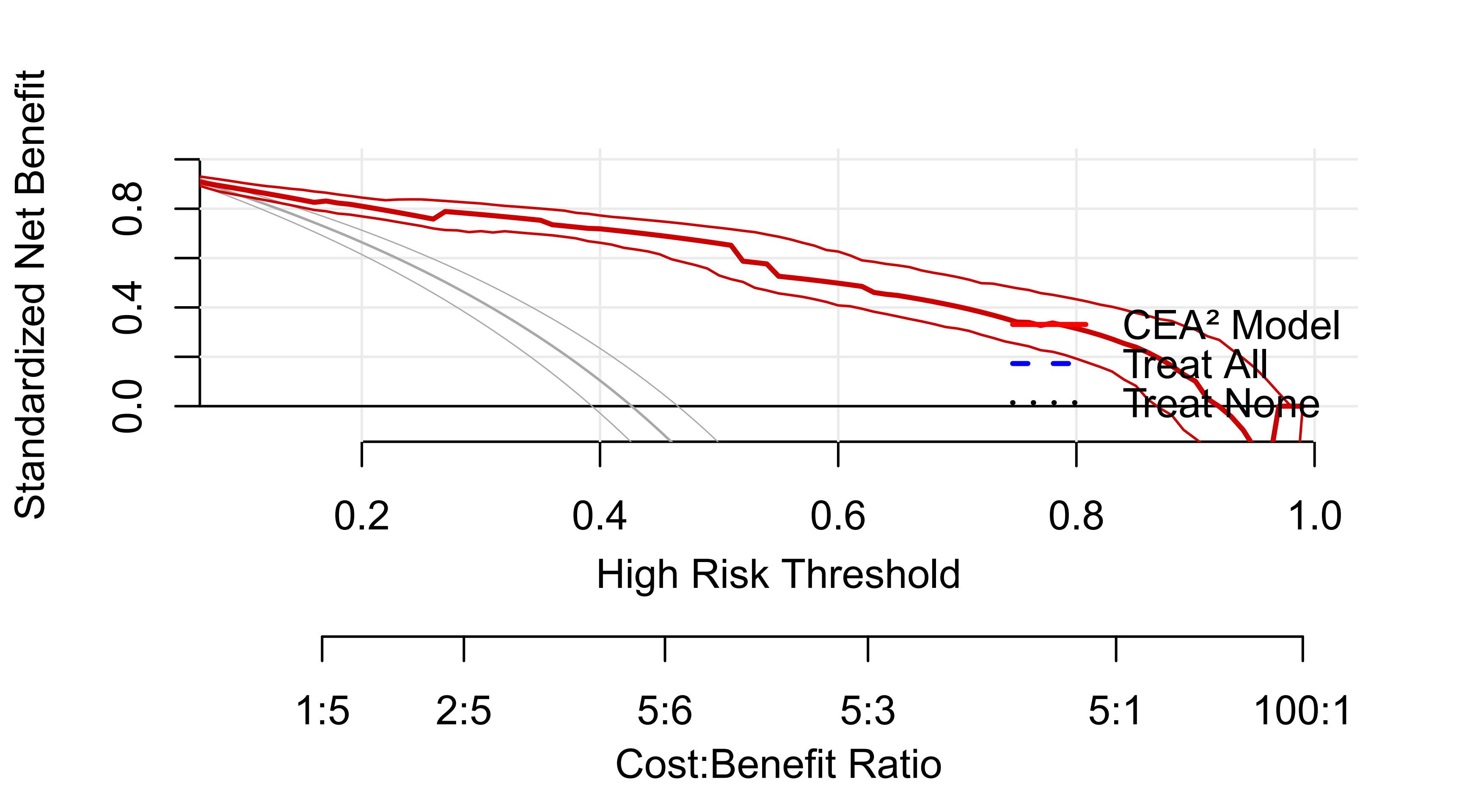

Supplement: Supplementary file 4 [file Image_4.tif]
