# Supplementary material for: A novel scale to predict acute anterior circulation large vessel occlusion stroke for community hospitals: result from the STRESS registry
Source: Front Neurol. 2026 Mar 27;17:1776311. doi: 10.3389/fneur.2026.1776311 (PMC13065725; doi:10.3389/fneur.2026.1776311)
Supplement: Supplementary file 5 [file Supplementary_file_1.docx]

**Supplement Table 1**. Comparison of baseline variables between LVOs and non-LVOs patients in derivation cohort.

SD: standard deviation；M: Median, Q_1_:1st Quartile, Q3:3st Quartile;AF, atrial

fibrillation; LVOs, large vessel occlusion strokes; LOC, level of consciousness;M1, M1 segment

of the middle cerebral artery; M2, M2 segment of the middle cerebral artery; NIHSS, national

1institutes of health stroke scale;CHD,coronary heart disease;ICA,intracranial internal carotid

Artery

| Variables | Non-LVOs (n =254) | LVOs (n = 152) | *P* |
| --- | --- | --- | --- |
| Age, year, Mean ± SD | 69.51 ± 13.22 | 69.09 ± 12.22 | 0.750 |
| Male, n (%) | 153 (60.24) | 93 (61.18) | 0.850 |
| Hypertension, n(%) | 155 (61.02) | 102 (67.11) | 0.219 |
| Diabetes mellitus, n(%) | 67 (26.38) | 36 (23.68) | 0.546 |
| CHD, n(%) | 21 (8.27) | 18 (11.84) | 0.237 |
| AF, n(%) | 25 (9.84) | 73 (48.03) | <.001 |
| Hyperlipidemia, n(%) | 100 (39.37) | 58 (38.16) | 0.808 |
| Hyperhomocysteinemia,n(%) | 45 (17.72) | 25 (16.45) | 0.743 |
| History of stroke, n(%) | 29 (11.42) | 22 (14.47) | 0.368 |
| Smoking, n(%) | 45 (17.72) | 29 (19.08) | 0.731 |
| Alcohol, n(%) | 23 (9.06) | 16 (10.53) | 0.626 |
| NIHSS score, M (Q₁, Q₃) | 4.00(2.00, 6.00) | 12.00 (9.00,16.00) | <.001 |
| A) LOC, M (Q₁, Q₃) | 0.00 (0.00, 0.00) | 1.00 (0.00, 1.00) | <.001 |
| B)LOC questions, M (Q₁,Q3) | 0.00 (0.00, 0.00) | 1.00 (0.00, 2.00) | <.001 |
| C) LOC commands, M(Q₁, Q₃) | 0.00 (0.00, 0.00) | 1.00 (0.00, 2.00) | <.001 |
| Gaze deviation, M (Q₁, Q₃) | 0.00 (0.00, 0.00) | 1.00 (0.00, 2.00) | <.001 |
| Visual field test, M (Q₁, Q₃) | 0.00 (0.00, 0.00) | 0.00 (0.00, 0.00) | >0.99 |
| Facial palsy, M (Q₁, Q₃) | 0.00 (0.00, 1.00) | 1.00 (1.00, 2.00) | <.001 |
| Motor left arm, M (Q₁, Q₃) | 0.00 (0.00, 1.00) | 0.00 (0.00, 4.00) | <.001 |
| Motor right arm, M (Q₁, Q₃) | 0.00 (0.00, 1.00) | 0.00 (0.00, 4.00) | <.001 |
| Motor left leg, M (Q₁, Q₃) | 0.00 (0.00, 1.00) | 0.00 (0.00, 4.00) | <.001 |
| Motor right leg, M (Q₁, Q₃) | 0.00 (0.00, 1.00) | 0.00 (0.00, 3.00) | <.001 |
| Limb ataxia, M (Q₁, Q₃) | 0.00 (0.00, 0.00) | 0.00 (0.00, 0.00) | >0.99 |
| Sensory, M (Q₁, Q₃) | 0.00 (0.00, 0.00) | 0.00 (0.00, 0.00) | 0.122 |
| Aphasia, M (Q₁, Q₃) | 0.00 (0.00, 0.00) | 1.00 (0.00, 3.00) | <.001 |
| Dysarthria, M (Q₁, Q₃) | 1.00 (0.00, 1.00) | 1.00 (0.00, 1.00) | 0.204 |
| Extinction and inattention,M (Q₁, Q₃) | 0.00 (0.00, 0.00) | 0.00 (0.00, 0.00) | >0.99 |
| Occlusion sites |  |  |  |
| ICA, n (%) | NA | 43（28.29） | NA |
| M1, n (%) | NA | 79（51.97） | NA |
| M2, n (%) | NA | 30（19.74） | NA |

**Supplement table 2.** Comparison of baseline variables between LVOs and non-LVOs patients in validation cohort.

LVOs,large vessle occlusion strokes; SD, standard deviation；M, Median, Q1,1st Quartile, Q3,3st Quartile;AF, atrial fibrillation; LVOs, large vessel occlusion strokes; LOC, level of consciousness;M1, M1 segment of the middle cerebral artery; M2, M2 segment of the middle cerebral artery; NIHSS, national institutes of health stroke scale;CHD,coronary heart disease;ICA,intracranial internal carotid Artery.

| Variables | Non-LVOs (n =254) | LVOs (n = 152) | *P* |
| --- | --- | --- | --- |
| Age, year, Mean ± SD | 70.12 ± 11.48 | 69.35 ± 12.23 | 0.563 |
| Male, n (%) | 85 (53.12) | 94 (60.26) | 0.201 |
| Hypertension, n(%) | 105 (65.62) | 96 (61.54) | 0.450 |
| Diabetes mellitus, n(%) | 35 (21.88) | 40 (25.64) | 0.431 |
| CHD, n(%) | 17 (10.62) | 22 (14.10) | 0.347 |
| AF, n(%) | 13 (8.12) | 73 (46.79) | <.001 |
| Hyperlipidemia, n(%) | 14 (8.75)  15 (9.38) | 20 (12.82)  23 (14.74) | 0.243  0.142 |
| Hyperhomocysteinemia,n(%) | 15 (9.38) | 23 (14.74) | 0.142 |
| History of stroke, n(%) | 52 (32.50) | 48 (30.77) | 0.741 |
| Smoking, n(%) | 25 (15.62) | 22 (14.10) | 0.704 |
| Alcohol, n(%) | 15 (9.38) | 14 (8.97) | 0.902 |
| NIHSS score, M (Q₁, Q₃) | 4.00 (3.00, 7.00) | 13.00 (8.00,18.00) | <.001 |
| A) LOC, M (Q₁, Q₃) | 0.00 (0.00, 0.00) | 1.00 (0.00, 1.00) | <.001 |
| B)LOC questions, M (Q₁,Q3) | 0.00 (0.00, 1.00) | 1.00 (0.00, 2.00) | <.001 |
| C) LOC commands, M(Q₁, Q₃) | 0.00 (0.00, 0.00) | 1.00 (0.00, 2.00) | <.001 |
| Gaze deviation, M (Q₁, Q₃) | 0.00 (0.00, 0.00) | 1.00 (0.00, 2.00) | <.001 |
| Visual field test, M (Q₁, Q₃) | 0.00 (0.00, 0.00) | 0.00 (0.00, 0.00) | >0.99 |
| Facial palsy, M (Q₁, Q₃) | 1.00 (0.00, 1.00) | 1.00 (1.00, 2.00) | <.001 |
| Motor left arm, M (Q₁, Q₃) | 0.00 (0.00, 1.00) | 0.00 (0.00, 4.00) | <.001 |
| Motor right arm, M (Q₁, Q₃) | 0.00 (0.00, 1.00) | 0.00 (0.00, 4.00) | <.001 |
| Motor left leg, M (Q₁, Q₃) | 0.00 (0.00, 1.00) | 0.00 (0.00, 3.00) | <.001 |
| Motor right leg, M (Q₁, Q₃) | 0.00 (0.00, 1.00) | 0.00 (0.00, 3.00) | <.001 |
| Limb ataxia, M (Q₁, Q₃) | 0.00 (0.00, 0.00) | 0.00 (0.00, 0.00) | >0.99 |
| Sensory, M (Q₁, Q₃) | 0.00 (0.00, 0.00) | 0.00 (0.00, 0.00) | 0.125 |
| Aphasia, M (Q₁, Q₃) | 0.00 (0.00, 0.00) | 1.00 (0.00, 3.00) | <.001 |
| Dysarthria, M (Q₁, Q₃) | 1.00 (0.00, 1.00) | 1.00 (0.00, 1.00) | 0.763 |
| Extinction and inattention,M (Q₁, Q₃) | 0.00 (0.00, 0.00) | 0.00 (0.00, 0.00) | >0.99 |
| Occlusion sites |  |  |  |
| ICA, n (%) | NA | 45（28.85）  80（51.28）  31（19.87） | NA |
| M1, n (%) | NA | 80(51.28）  31（19.87） | NA |
| M2, n (%) | NA | 31（19.87）  31（19.87） | NA |

**Supplement Table 3**. Comparison of baseline variables between derivation and validation cohorts.LVOs,large vessle occlusion strokes;SD, standard deviation；M, Median, Q_1_:1st Quartile, Q3:3st Quartile;AF, atrial fibrillation; LVOs, large vessel occlusion strokes; LOC, level of consciousness;M1, M1 segment of the middle cerebral artery; M2, M2 segment of the middle cerebral artery; NIHSS, national institutes of health stroke scale;CHD,coronary heart disease;ICA,intracranial internal carotid Artery

| Variables | Derivation cohort(n=406) | Validation cohort (n=316) | | *P value* |
| --- | --- | --- | --- | --- |
| Age, year, Mean ± SD | 69.59±11.77 | | 69.85 ± 11.47 | 0.938 |
| Male, n (%) | 260 (64.04) | | 161 (50.95) | 0.001 |
| Hypertension, n(%) | 258(63.55) | | 201(63.61) | 0.987 |
| Diabetes mellitus, n(%) | 103(25.37) | | 76(24.05) | 0.684 |
| CHD, n(%) | 38 (9.36) | | 38 (12.03) | 0.247 |
| AF, n(%) | 98(24.14) | | 86(27.2) | 0.347 |
| Hyperlipidemia, n(%) | 158(38.92) | | 100(31.65) | 0.052 |
| Hyperhomocysteinemia,n(%) | 70(17.24) | | 34(10.76) | 0.019 |
| History of stroke, n(%) | 51 (12.6) | | 38 (12.0) | 0.828 |
| Smoking, n(%) | 74(18.2) | | 47 (14.9) | 0.231 |
| Alcohol, n(%) | 39(9.6) | | 29 (9.2) | 0.845 |
| NIHSS score, M (Q₁, Q₃) | 6.00(300, 11.00) | | 8.00(4.00,13.00) | <.001 |
| A) LOC, M (Q₁, Q₃) | 0.00 (0.00, 1.00) | | 0.00 (0.00, 1.00) | 0.030 |
| B)LOC questions, M (Q₁,Q3) | 0.00 (0.00, 1.00) | | 0.00 (0.00, 2.00) | <.001 |
| C) LOC commands, M(Q₁, Q₃) | 0.00 (0.00, 1.00) | | 1.00 (0.00, 1.00) | 0.125 |
| Gaze deviation, M (Q₁, Q₃) | 0.00 (0.00,1.00) | | 0.00 (0.00,1.00) | 0.020 |
| Visual field test, M (Q₁, Q₃) | 0.00 (0.00, 0.00) | | 0.00 (0.00, 0.00) | 1.000 |
| Facial palsy, M (Q₁, Q₃) | 1.00 (0.00, 1.00) | | 1.00 (0.00, 2.00) | <.001 |
| Motor left arm, M (Q₁, Q₃) | 0.00 (0.00, 2.00) | | 0.00 (0.00, 2.00) | 0.229 |
| Motor right arm, M (Q₁, Q₃) | 0.00 (0.00, 2.00) | | 0.00 (0.00, 2.00) | 0.127 |
| Motor left leg, M (Q₁, Q₃) | 2.00 (0.00,3.00) | | 2.00 (1.00, 4.00) | 0.009 |
| Motor right leg, M (Q₁, Q₃) | 0.00 (0.00, 1.00) | | 0.00 (0.00, 2.00) | 0.355 |
| Limb ataxia, M (Q₁, Q₃) | 0.00 (0.00, 1.00) | | 0.00 (0.00, 2.00) | 0.008 |
| Sensory, M (Q₁, Q₃) | 0.00 (0.00, 2.00) | | 0.00 (1.00,3.00) | 0.007 |
| Aphasia, M (Q₁, Q₃) | 0.00 (0.00, 0.00) | | 1.00 (0.00, 0.00) | 1.000 |
| Dysarthria, M (Q₁, Q₃) | 0.00 (0.00, 0.00) | | 0.00 (0.00, 0.00) | 0.181 |
| Extinction and inattention,M (Q₁, Q₃) | 0.00 (0.00, 0.00) | | 0.00 (0.00, 1.00) | 0.001 |
| Occlusion sites of LVOs |  | |  | 0.016 |
| ICA, n (%) | 43（10.59） | | 45（14.24） |  |
| M1, n (%) | 79（19.46） | | 80（25.32） |  |
| M2, n(%) | 30（7.39） | | 31（9.81） |  |

**Supplement Table 4. Multivariable logistic regression analysis for predictors of anterior circulation LVOs.**

LVOs,large vessle occlusion strokes;OR,odd ratio;CI,confidence interval

| **Variable** | **β** | **SE** | **OR** | **95% CI** | ***P* value** |
| --- | --- | --- | --- | --- | --- |
| Intercept | -2.69 | 0.29 | 0.07 | 0.04–0.12 | <0.001 |
| Level of consciousness | 1.27 | 0.30 | 3.54 | 1.97–6.39 | <0.001 |
| Gaze deviation | 1.84 | 0.33 | 6.28 | 3.31–12.26 | <0.001 |
| Arm weakness | 1.48 | 0.31 | 4.39 | 2.44–8.15 | <0.001 |
| Atrial fibrillation | 1.22 | 0.32 | 3.38 | 1.81–6.42 | <0.001 |

**Supplement Table 5. Bootstrap internal validation.**AUC,area under the curve.CI,confidence interval

| **indicators** | **Value** | **95% CI** |
| --- | --- | --- |
| apparent AUC | 0.881 | 0.856–0.906 |
| average optimism | 0.0045 | - |
| optimism-corrected AUC | 0.876 | 0.874–0.883 |

Supplement Table 6.Multicollinearity assessment:Pearson correlation matrix among candidate predictors.VIF,Pearson correlation coefficients and variance inflation factors.

| **Variable** | **VIF** | **LASSO Coefficient (λ.1se)** |
| --- | --- | --- |
| Level of consciousness | 1.57 | 0.413 |
| Gaze deviation | 1.73 | 1.126 |
| Arm weakness | 1.42 | 0.571 |
| Atrial fibrillation | 1.25 | 0.472 |
| Facial palsy | 1.47 | 0.200 |
| Aphasia | 1.59 | 0.635 |

Supplement Table 7. Sensitivity analysis: performance comparison of models with and without

atrial fibrillation.AUC,area under the curve

| **Model** | **AUC (95% CI)** | **Optimal threshold** | **Sensitivity (%)** | **Specificity (%)** | ***P* value*** |
| --- | --- | --- | --- | --- | --- |
| Full model (with AF) | 0.881 (0.847–0.915) | 0.474 | 81.8 | 85.4 | – |
| Reduced model (without AF) | 0.867 (0.831–0.902) | 0.323 | 72.3 | 87.9 | 0.005 |
| * DeLong test for AUC comparison. |  |  |  |  |  |

**Table 8. Stability analyses: bootstrap selection frequency and robust standard errors.**

| **Variable*** | **Bootstrap retention (%)** | **Original OR (95% CI)** | **Robust SE OR (95% CI)** | **Original P** | **Robust P** |
| --- | --- | --- | --- | --- | --- |
| Level of consciousness | 87.4 | 2.38 (1.26–4.45) | 2.38 (1.22–4.61) | 0.007 | 0.011 |
| Gaze deviation | 99.6 | 4.22 (2.14–8.49) | 4.22 (1.98–8.97) | <0.001 | 0.0002 |
| Arm weakness | 99.0 | 3.18 (1.67–6.17) | 3.18 (1.71–5.90) | 0.0005 | 0.0002 |
| Atrial fibrillation | 97.2 | 3.05 (1.62–5.81) | 3.05 (1.52–6.09) | 0.0006 | 0.0016 |
| Facial palsy | 79.2 | 2.13 (1.09–4.26) | 2.13 (1.02–4.45) | 0.029 | 0.043 |
| Aphasia | 94.6 | 3.31 (1.63–6.80) | 3.31 (1.51–7.26) | 0.001 | 0.003 |

OR = odds ratio; CI = confidence interval; Robust SE model used HC3 standard errors.*
