# Supplementary material for: A novel scale to predict acute anterior circulation large vessel occlusion stroke for community hospitals: result from the STRESS registry
Source: Front Neurol. 2026 Mar 27;17:1776311. doi: 10.3389/fneur.2026.1776311 (PMC13065725; doi:10.3389/fneur.2026.1776311)
Supplement: Supplementary file 6 [file Supplementary_file_2.doc]

SFigure legend:

**SFigure 1.Decision curve analysis comparing the full logistic model and the simplified CEA² score.**CEA^2^,Consciousness-Eye-Arm-Atrial Fibrillation

**SFigure 2.****Cross-validation plot for LASSO regression.** The binomial deviance (mean ± standard error) is plotted against log(λ). LASSO,least absolute shrinkage and selection operator regression

**SFigure 3. Calibration plot for the CEA² model.**CEA^2^,Consciousness-Eye-Arm-Atrial Fibrillation

**SFigure 4. Decision curve analysis for the CEA² model.** CEA^2^,Consciousness-Eye-Arm-Atrial Fibrillation
